# An Integrative Toxicological Assessment of the Herbicide Tebuthiuron: Elucidating Biochemical and Behavioral Responses in Developing Zebrafish

**DOI:** 10.3390/jox16050166

**Published:** 2026-09-03

**Authors:** Giovane Ferreira, Amany Sultan, Fatma Ceren Kirgiz, Jacqueline Cristina Gutierrez, Evelyn C. López González, Christopher J. Martyniuk

**Affiliations:** 1 Engineering School of São Carlos, University of São Paulo, São Carlos 13566-590, SP, Brazil; giovane.fer@usp.br; 2 Center for Environmental and Human Toxicology, Department of Physiological Sciences, College of Veterinary Medicine, University of Florida, Gainesville, FL 32611, USA; amanysultan2025@gmail.com (A.S.); fatmacerenkirgiz@gmail.com (F.C.K.); j.gutierrez1@ufl.edu (J.C.G.); evelynclg@gmail.com (E.C.L.G.); 3 Animal Health Research Institute, Agriculture Research Centre, Giza 12619, Egypt; 4 Department of Pharmacology and Toxicology, Faculty of Veterinary Medicine, Hatay Mustafa Kemal University, Hatay 31060, Türkiye; 5 Laboratorio de Ecotoxicología, Facultad de Humanidades y Ciencias, Universidad Nacional del Litoral-Consejo Nacional de Investigaciones Científica y Técnicas (CONICET-UNL), Santa Fe 3000, Argentina; 6 Interdisciplinary Program in Biomedical Sciences Neuroscience, UF Genetics Institute, Gainesville, FL 32611, USA

**Keywords:** zebrafish, tebuthiuron, ecotoxicology, oxidative stress, neurotoxicity, locomotor activity

## Abstract

Pesticides represent a significant threat to aquatic ecosystems due to their persistence and widespread use in agricultural areas, altering environments and exerting adverse effects on non-target organisms. Tebuthiuron is a phenylurea herbicide extensively used as an agrochemical to control pests and weeds in various crops, which often leads to contamination of aquatic environments. Despite its high water solubility and relatively long half-life in soil, studies on Tebuthiuron toxicity in fishes at environmental concentrations are limited. This study aimed to unravel the toxicity mechanisms of Tebuthiuron using the zebrafish model. Zebrafish embryos were exposed to Tebuthiuron (one concentration of either 0.1, 10, 1000 and 5000 µg/L) for 5 days and assessed for hatchability, heart rate, locomotor activity, oxygen reactive species, apoptosis and gene expression. There was no change in frequency of hatch, heart rate, or apoptosis. However, behavioral changes were noted, with hyperactivity in zebrafish larvae during the first light (at 10 µg/L), second light (at 10 and 1000 µg/L) and third dark (10 µg/L) periods of the Visual Motor Response assay. Biochemically, a significant depletion of basal ROS levels was observed at 1000 µg/L. At the molecular level, downregulation of oxidative stress-related genes (*cat*, *sod1*, and *sod2*) in larval fish was noted with exposure to 10 and 5000 µg/L Tebuthiuron, suggesting a depletion of antioxidant enzymes. In addition, some neurotoxicity-related genes were downregulated, such as acetylcholinesterase (*ache*), while some genes were upregulated, like microtubule-associated protein tau b (*maptb*) and synapsin 2 alpha (*syn2a*), with 5000 µg/L Tebuthiuron exposure. In conclusion, Tebuthiuron induces sublethal toxicity characterized by irregular, concentration-specific disruptions to photomotor behavior, basal ROS levels, and transcriptional markers of oxidative stress and synaptic function, even in the absence of acute morphological defects. Future research should prioritize functional assays targeting mitochondrial bioenergetics and antioxidant enzyme activities, alongside evaluations of later developmental stages, to fully elucidate the long-term ecological risks of phenylurea herbicides to non-target aquatic species.

## 1. Introduction

Since 2008, Brazil has been the leading consumer of pesticides globally, followed by the United States of America. Over the last decade, Brazil’s pesticide consumption increased by 190% compared to previous years, a growth rate of more than double the global market’s 93% increase during the same period [1]. In the 2010/2011 season, Brazilian pesticide consumption reached 936,000 tons, resulting in $8.5 billion in financial transactions [2]. In 2014, the use of other pesticides accounted for the highest proportion of total pesticides (53.84%), followed by herbicides (25.10%), fungicides and bactericides (12.06%), insecticides (7.50%), plant growth regulators (1.24%), and others [3]. Determining how these chemicals enter aquatic systems and their associated risks to aquatic species is therefore paramount for their safe application and use.

Tebuthiuron (TBH), chemically named N-[5-(1,1-dimethylethyl)-1,3,4- thiadiazol-2-y1]-N,N′-dimethylurea (C_9_H_16_N_4_OS), has a molecular weight of 228.314 g/mol and a log Kow of 1.79 [4,5]. TBH is an herbicide employed for brush control in pastures, rangelands, rights-of-way, and industrial sites. This phenylurea herbicide, commercially introduced in the mid-1970s [6], enters weeds via root and foliar absorption. It subsequently kills weeds by inhibiting electron transport, primarily at the reducing side of photosystem II during photosynthesis [7]. The carryover of biotoxins through residual TBH leaching can lead to contamination and biodiversity loss due to food chain bioaccumulation or environmental exposure [8].

TBH exhibits soil persistence ranging from 11 to 25 months, potentially extending up to 7.2 years, and has a DT50 (Degradation Half-Life) ranging from 16 to 20 days. With high water solubility (2500 mg/L), TBH is considered relatively persistent and possesses a high leaching potential [9,10]. As such, it has been detected in freshwater environments at concentrations ranging from 10 to 291 ng/L [11]. For instance, TBH was detected in Pardo River water, São Paulo, Brazil reaching a maximum concentration of 1.02 μg/L [12]. It was also measured in the Mogi Guaçu River Basin in Brazil at an alarming maximum concentration of 6.4 μg/L, with a detection frequency of 100% [13].

Behavior is a key component of survival and fitness in aquatic organisms, influencing essential ecological processes such as foraging, predator avoidance, reproduction, habitat selection, and social interactions. Because behavioral responses integrate physiological, neurological, and ecological processes, they are considered sensitive indicators of environmental disturbance. Importantly, behavioral alterations may occur at contaminant concentrations below those causing mortality or evident physiological impairment, providing an early warning of sublethal effects. Consequently, behavioral endpoints have been increasingly incorporated into studies as ecologically relevant tools for assessing the impacts of environmental contaminants on aquatic species and populations [14]. In the context of TBH, these sublethal disruptions are highly relevant; recent screenings have linked this herbicide to acetylcholinesterase (AChE) inhibition, altered embryonic tail coiling, and distinct late-stage larval hyperactivity specifically during dark photoperiods in zebrafish [15].

While some studies, based on theoretical criteria and mathematical models, describe the susceptibility of groundwater to TBH contamination, research conducted in laboratory and field settings using soil columns and soil and water sample collections indicate lower leaching of this herbicide [16]. However, questions remain regarding its contamination potential and effects on aquatic organisms. In the USA, a maximum of 4.5% of the TBH applied to watersheds (2.24 kg a.i./ha) was determined to run off into adjacent water bodies. After watershed treatment, TBH concentration in receiving lotic waters exhibited a range from nondetectable to 83 μg/L [17]. TBH therefore is present in aquatic ecosystems, posing a risk of adverse exposures to aquatic species.

Within this context of environmental exposure, the zebrafish (*Danio rerio*) serves as a premier vertebrate model for studying sublethal developmental toxicity. Early life stages (e.g., up to 5 days post-fertilization) are particularly vulnerable to environmental toxicants, as interference with rapid morphological and neurological maturation during this window provides highly sensitive indicators of sublethal disruption [18].

While the acute toxicity and morphological impacts of various photosystem inhibitor herbicides are well-documented, the sublethal molecular mechanisms of TBH remain poorly understood. A broader look at phenylurea toxicity in aquatic vertebrates reveals that this class of herbicides profoundly disrupts endocrine systems, particularly the hypothalamus–pituitary–gonadal (HPG) and thyroid (HPT) axes, and alters extensive reproductive, oxidative, and metabolic networks [19]. In line with these sublethal perturbations, recent studies indicate that structurally related phenylurea herbicides, such as linuron, induce severe metabolic depression and neurotoxic hypoactivity in early staged zebrafish [20]. Moreover, initial behavioral screening of TBH have reported acetylcholinesterase (AChE) inhibition and late-stage larval hyperactivity [15]. This indicates that TBH may act through complex, non-linear neurodevelopmental and bioenergetic cascades that have yet to be fully elucidated.

Given Brazil’s leading and increasing pesticide consumption, combined with the persistence of TBH in international watersheds, it is reasoned that this chemical represents a significant environmental concern. To address ecotoxicological knowledge gaps, this study utilizes the zebrafish model to conduct an integrative assessment of TBH toxicity. Based on previous research, we hypothesized that zebrafish would show impaired behavioral responses and evidence of oxidative stress at high concentrations. By evaluating a suite of behavioral, biochemical, and transcriptome responses, this research aims to define the precise sublethal mechanisms and neurodevelopmental risks this herbicide poses to aquatic ecosystems.

## 2. Materials and Methods

### 2.1. Chemical Preparation

Tebuthiuron (TBH) [PESTANAL^®^, analytical standard (Sigma Aldrich, St. Louis, MO 63178, USA; CAS Number: 34014-18-1; purity ≥ 99%)] stock solutions were prepared and added to Embryo Rearing Media (ERM) containing the zebrafish embryos. The selected TBH concentrations (0.1, 10, 1000 and 5000 μg/L) were chosen to encompass a broad exposure range, including concentrations within the range reported [15] in aquatic environments and higher concentrations commonly used in ecotoxicological assessment. Environmental monitoring studies have detected TBH in surface water, with concentrations up to 1.02 μg/L reported in the Pardo River and Mogi Guaçu River basins in Brazil [12]. The higher concentration was included to evaluate concentration-dependent responses and potential toxicological mechanisms under elevated exposure scenarios. To ensure chemical stability and prevent the buildup of metabolic waste, fresh stocks were prepared daily to replace the corresponding volume during media renewal.

### 2.2. Husbandry and Egg Production of Zebrafish

Adult zebrafish (AB × Tübingen, *Danio rerio*) were maintained under controlled laboratory conditions. Details on breeding and water quality have been outlined previously, and these studies outline the husbandry conditions [21,22]. Adult breeders are monitored for humane endpoints that include notable infections, impaired swimming behavior, or damaged tissues. Breeders are between 6 months and 1 year old. Staging of embryos and general husbandry protocols followed established guidelines. The Institutional Animal Care and Use Committee approved all experiments (IACUC202100000080, Approval date: 3 October 2022).

### 2.3. TBH Exposure Design

Fertilized and normally developing embryos were selected at ~6 h post-fertilization (hpf) and randomly assigned to experimental groups: an ERM negative control and a range of TBH concentrations. Four experiments were conducted with approximately 350 larval fish used per experiment. Four to six beakers (25 mL Pyrex glass beakers) per treatment group were used depending on the number of eggs available from each breeding event, and each beaker contained 15–20 embryos in 10 mL of the respective test solution. The beaker was the biological unit. Beakers were maintained in an incubator at 27 ± 1 °C. A 90% media renewal was performed daily for a continuous 5-day exposure period. Embryos and larvae were assessed daily using an EVOS™ FL Auto Imaging System (Thermo Fisher Scientific, Waltham, MA, USA) to record mortality throughout the continuous 120 h exposure period. Hatch rates and documented morphological deformities (spinal lordosis, caudal tail malformations, and yolk sac/pericardial edema) were recorded.

Heart rate analysis was conducted at 5 days post-fertilization (dpf) using 10 randomly selected individuals per experimental group (2 fish from each 5 beakers in the experimental group). Heartbeats were counted under the EVOS™ FL Auto Imaging System for a period of 10 s per larva, and the resulting values were multiplied by six to determine the total beats per minute (bpm). Prior to storing embryos for assays, fish were euthanized using buffered MS-222 (250 mg/L) (Syndel’s Syncaine^®^, Ferndale, WA, USA) Fish Anesthetic, pooled within a 1.7 mL tube (1 beaker = 1 biological replicate = 1 pool), immediately frozen in liquid nitrogen, and placed into a −80 freezer for ROS measurements or RNA extraction.

### 2.4. Visual Motor Response Test

Behavioral responses were assessed in 5 dpf larvae utilizing a DanioVision™ Observation Chamber (Noldus Information Technology, Leesburg, VA, USA). Individual larvae were transferred into a 96-well plate containing 200 μL of ERM per well. Following a 2 h acclimation period to minimize handling stress, a 50 min Visual Motor Response (VMR) protocol was initiated, consisting of alternating 10 min dark and light phases. The assay began with a dark period to specifically evaluate photokinesis. Total distance moved (mm) was tracked continuously using EthoVision^®^ XT software (Version 12, Noldus Information Technology, Wageningen, The Netherlands). Trajectories demonstrating poor tracking fidelity, defined objectively as continuous tracking loss exceeding 10% of the total assay duration or erratic, impossible velocity spikes indicating software detection errors, were systematically excluded based on manual inspection of the tracking files. Three experiments were conducted. To accurately combine the data and account for baseline inter-assay variability across the separate runs, a relative normalization procedure was applied. For each independent run, the mean distance moved by the ERM control group within each specific time bin was established as the baseline (standardized to a value of 1). The relative distance moved for each individual larvae (TBH-treated) was subsequently calculated as a relative measure compared to this within-run baseline. For example, the ERM group was averaged to a normalized distance moved equal to 1, and all other groups were relative to the ERM group. Standard operating procedures have been published previously for our VMR assay [23].

### 2.5. Reactive Oxygen Species

At 5 dpf, surviving larvae from each treatment group were pooled (*n* = 3–5 biological replicates per group), transferred to microcentrifuge tubes, and homogenized in 200 μL of ice-cold phosphate-buffered saline (PBS). Homogenates were centrifuged at 12,000× *g* for 20 min at 4 °C. For ROS quantification, 20 μL of the supernatant was incubated with 2′,7′-Dichlorofluorescin Diacetate (H2-DCFDA) in a black fluorescence microplate for 30 min at 37 ± 1 °C in the dark. Samples were added to the microplate in triplicates. Fluorescence intensity was measured (excitation 485 nm; emission 520 nm). Final ROS levels were normalized to total protein content (μg/mL) using a BCA assay.

### 2.6. Acridine Orange Staining

Apoptosis was evaluated using Acridine Orange (AO) staining. Surviving 5 dpf larvae (n = 17–22 larvae/group) were incubated in 2 μg/mL of AO (CAS 65-61-2, Sigma-Aldrich, Burlington, MA, USA) in ERM for 30 min in the dark at 28 °C. Larvae were subsequently rinsed three times and imaged using a fluorescence microscope (EVOS TM Fl Auto Imaging System (ThermoFisher, Scientific, Waltham, MA, USA) with a GFP filter. The intensity and localization of the fluorescent signal were quantified using the histogram tool of the Image J software (v1.54t, released 16 May 2026; Bethesda, MD, USA) to determine tissue-specific apoptosis.

### 2.7. Real-Time PCR Analysis

Total RNA was extracted from pooled 5 dpf larval samples using 750 μL TRIzol^®^ Reagent (Life Technologies, Carlsbad, CA, USA) as per the manufacturer’s protocol. Samples were assessed for RNA concentration using the Qubit^®^Fluorometer (#Q33216, Invitrogen, Carlsbad, CA, USA). Complementary DNA (cDNA) synthesis was done using ~750 ng of RNA and ABScript Neo RT Master mix with gDNA remover (Abclonal Technology, Woburn, MA, USA, CAT. No. RK20433) in a final sample volume of 20 μL. Samples were placed into a T100™ Thermal Cycler (BioRad, Hercules, CA, USA) to synthesize cDNA. The cDNA was generated using the following steps: 37 °C for 2 min, 55 °C for 15 min, 85 °C for 5 min, and 4 °C for 5 min. Prior to real-time PCR, cDNA stocks were diluted 1:25 in RNase–DNase-free water. Quantitative real-time PCR was conducted using a fluorescence-based master mix. Real-time PCR methods followed those of our previous studies [20,21]. The relative expression levels of target genes were normalized to the geometric mean of three established housekeeping genes (Ribosomal subunit 18, *rps18*; Ribosomal subunit L13a, and beta-actin, *bactin*). Final relative mRNA abundance among the experimental groups was calculated. Normalized expression ∆∆Cq was calculated as the relative quantity of gene(s) of interest normalized to the relative quantity of the reference gene(s) across samples. The primer sequences used in this study are provided in the Supplemental Methods, Table S1 [23,24,25,26,27,28,29,30,31,32,33,34,35,36].

### 2.8. Statistical Analysis

Statistical analysis and graphing were conducted using GraphPad Prism V9.4 (GraphPad Software, San Diego, CA, USA). A Log-rank (Mantel–Cox) test was employed to analyze survival and hatch curves to compare differences between each TBH concentration and the ERM control over time. Groups being compared are those with different concentrations of TBH to ERM control (no chemical). ROS data were assessed for normality using a Shapiro–Wilk test; normally distributed data were analyzed via a One-Way ANOVA followed by a Dunnett’s multiple comparisons test, whereas non-normally distributed data were log-transformed prior to analysis (log10). 

VMR data within discrete time bins were logarithmically transformed then analyzed utilizing a One-Way ANOVA test followed by a post hoc Dunnett’s multiple comparisons test to compare each treatment group with the control (ERM), as the study was specifically designed to evaluate treatment-related effects relative to the control condition. Dunnett’s test is the recommended post hoc test for comparisons between the control group and each of the treatments. VMR data were normalized to the ERM group for each independent run. The ERM group was standardized to a value of 1 and each treatment was subsequently compared in a relative manner. Simple linear regression and One-Way ANOVA followed by a Dunnett’s post hoc test were conducted to evaluate gene expression variance across treatments. Because this study employed a targeted molecular approach focusing on a predefined subset of marker genes rather than global transcriptomic profiling, expression data were analyzed independently for each target transcript without a formal false discovery rate (FDR) correction. While this approach increases the probability of false-positive findings, it was chosen to maximize sensitivity for detecting targeted developmental disruptions. The significance of difference was determined at *p* < 0.05.

## 3. Results

### 3.1. Survival and Deformity

**Figure 1:**
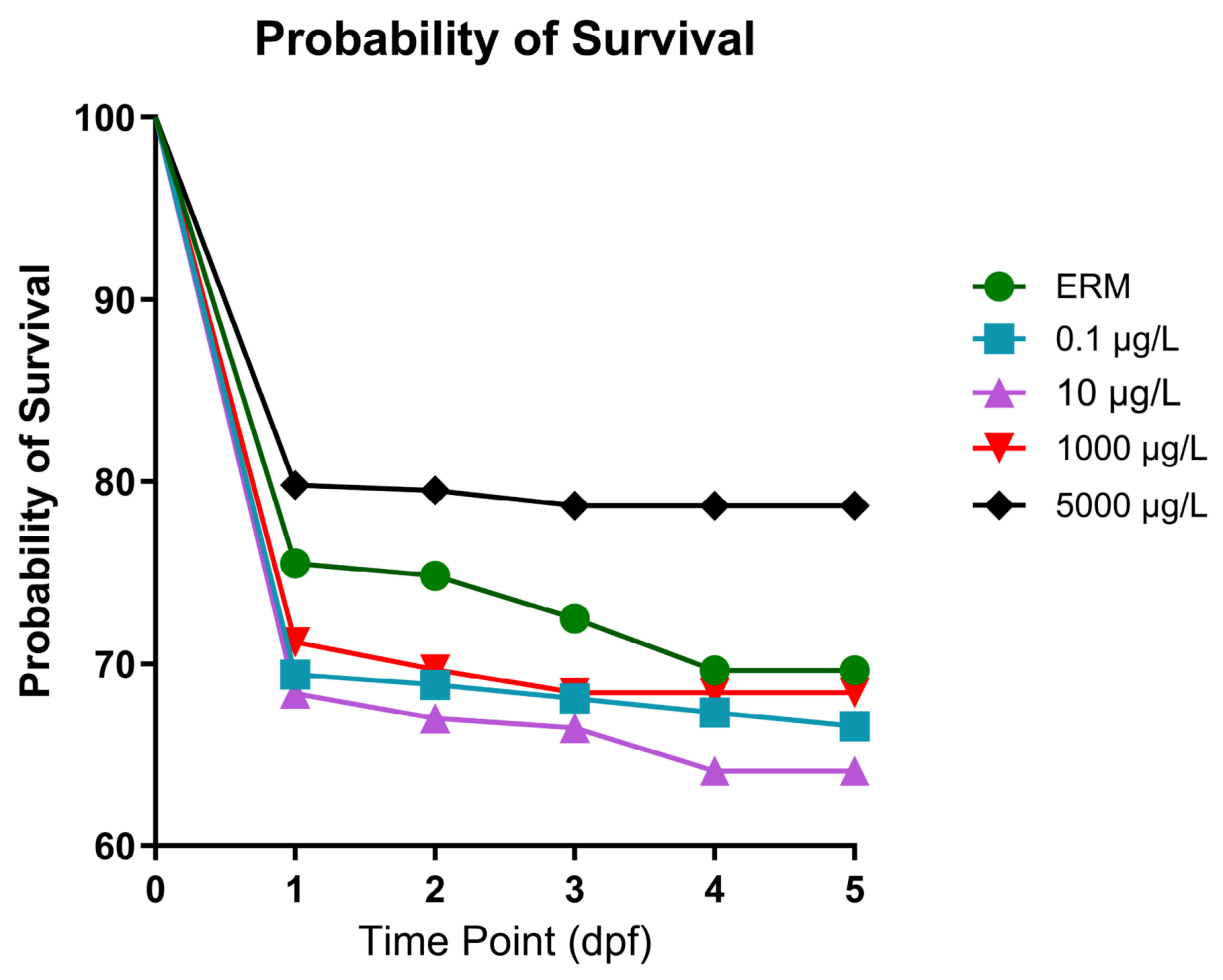
Survival of zebrafish embryos and larvae (0–5 dpf) exposed to TBH. Data are presented as a cumulative percentage of survival. No statistically significant differences were observed between the control (ERM) and treated groups (*p* > 0.05; Log-rank Mantel–Cox test).

Some initial mortality was observed within the first 24 h across all groups, including the ERM control. This was initially due to non-viable eggs, which stabilized after 24 h. Exposure to TBH at concentrations up to 5000 μg/L did not decrease in survival compared to the control group (Log-rank test, *p* > 0.05) (Figure 1). No treatment induced lower survival relative to the controls. This suggests that TBH does not induce acute lethal toxicity in developing zebrafish at the tested concentrations up to 5000 μg/L.

**Figure 2:**
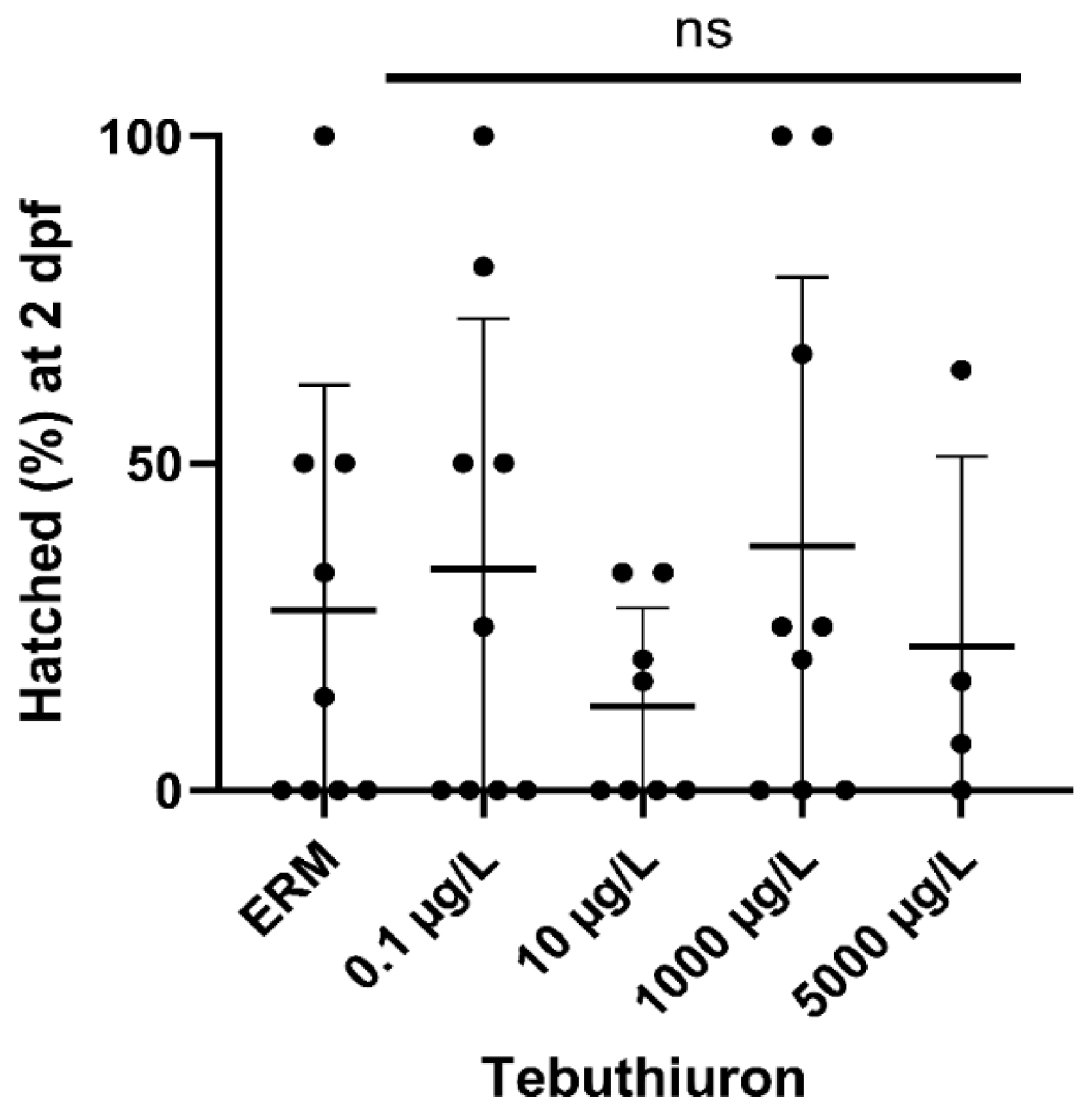
Hatching success of zebrafish embryos exposed to TBH from 0 to 120 hpf. Data represents the cumulative percentage of successfully hatched embryos per treatment group. Statistical significance was evaluated using One-Way ANOVA followed by a Dunnett’s test. No significant delays in hatching were observed across the tested concentrations (*p* > 0.05). ns = not significant.

**Figure 3:**
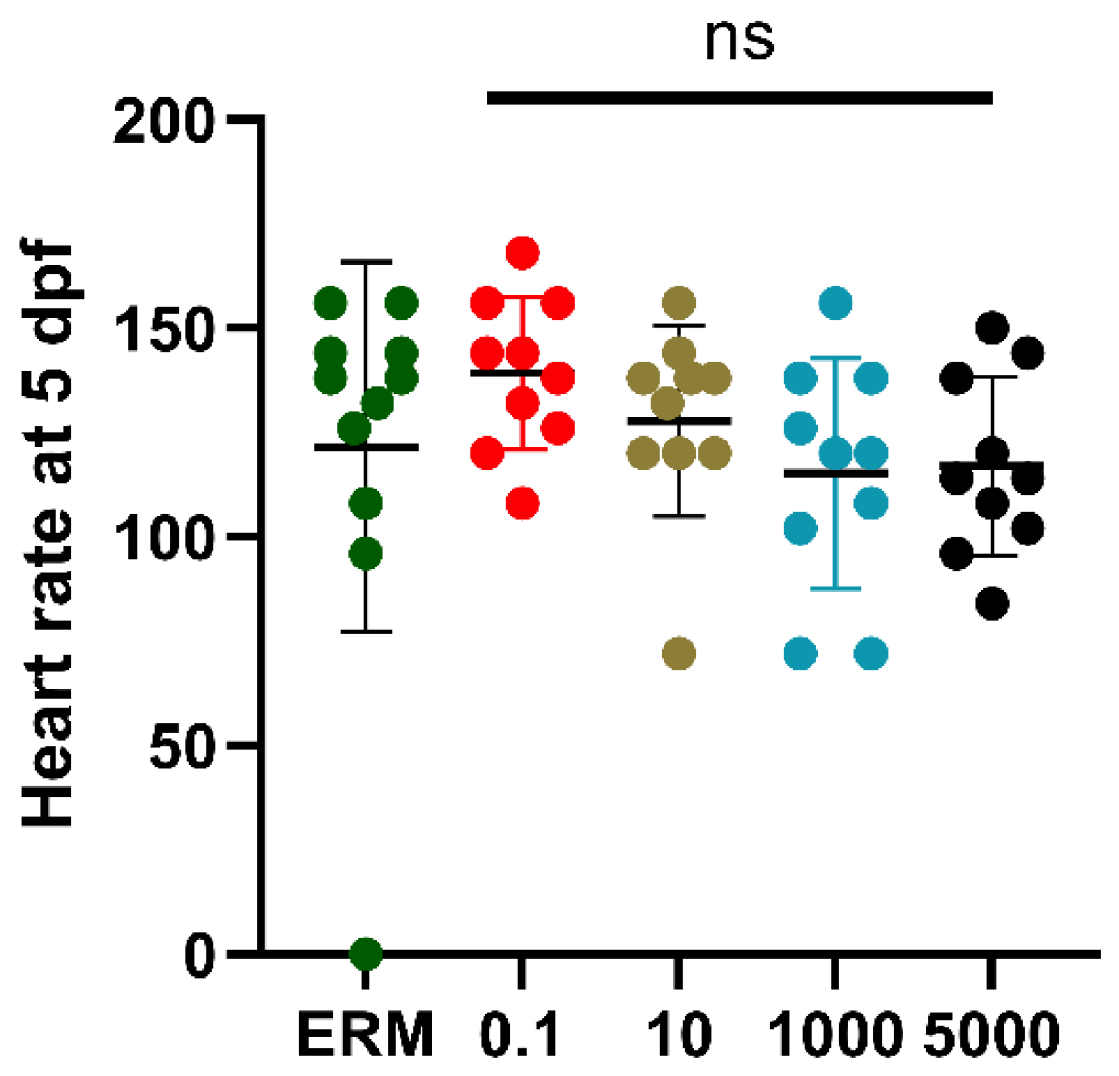
Heart rate in zebrafish larvae (5 dpf) after exposure to TBH (µg/L). Heart rate is expressed as beats per minute (bpm). Data are presented as mean ± SD (horizontal line and vertical line, respectively) (*n* = 10 fish per treatment). Each circle is an individual fish. No significant differences were detected between the control and treated groups (*p* > 0.05; One-Way ANOVA). ns = not significant.

Sporadic malformations, including instances of severe spinal lordosis, pericardial edema, and yolk sac edema were documented. However, these deformities were rare (less than 5%) across all groups. TBH exposure did not induce significant alterations in hatching rates (F_(4,34)_ = 0.67, *p* = 0.62) (Figure 2) across the tested concentrations (*p* > 0.05). Heart rate was not affected by exposure to TBH up to 5000 µg/L (F_(4,46)_ = 1.14, *p* = 0.35) (Figure 3).

### 3.2. TBH Induces Hyperactivity in Zebrafish Larvae

**Figure 4:**
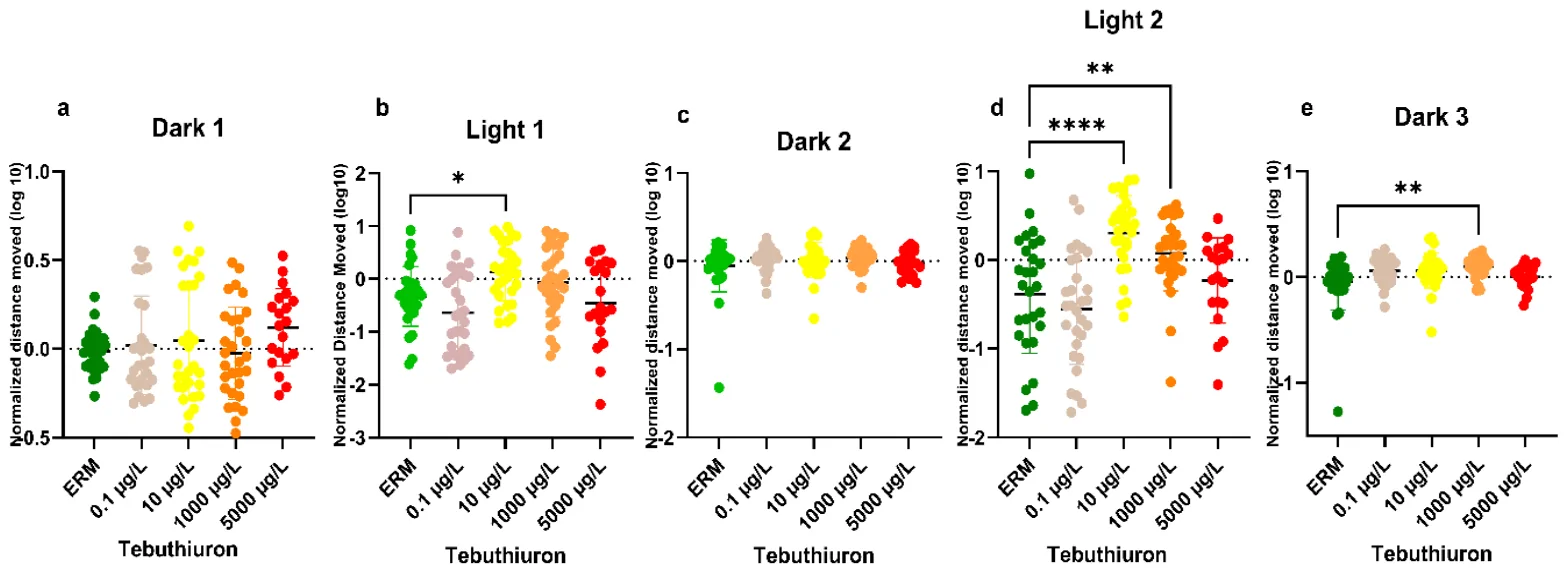
Effects of TBH on zebrafish larval (5 dpf) locomotor activity during the Visual Motor Response (VMR) assay. Total distance moved (mm) (log10 transformed) was recorded during alternating 10 min dark and light phases following a 2 h acclimation period. (**a**) Dark period 1, (**b**) Light period 1, (**c**) Dark period 2, and (**d**) Light period 2, (**e**) Dark period 3. Data are presented as mean ± SD (*n* = 133–135 larvae per treatment). Three independent experiments were conducted, and data were normalized to a value = 1 for the ERM group. All other treatments are presented as movement relative to ERM control. Asterisks indicate significant differences between the control and treated groups (* *p* < 0.05; ** *p* < 0.01; **** *p* < 0.0001), as determined by a One-Way ANOVA test followed by Dunnett’s post hoc test for discrete time bins.

Behavioral responses are presented in Figure 4a–e. During the initial sudden transition to light (Light 1) (F_(4,133)_ = 6.24, *p* < 0.0001), which naturally induces hypoactivity in healthy zebrafish, larvae exposed to 10 μg/L TBH exhibited hyperactivity (*p* = 0.03), in line with the Light 2 phase (F_(4,133)_ = 12.4, *p* < 0.0001), which showed a high hyperactive response in zebrafish exposed to both 10 μg/L (*p* < 0.0001) and 1000 μg/L (*p* < 0.005). The total distance moved in these groups was significantly increased compared to the ERM control (F_(4,133)_ = 6.241; *p* = 0.0001). In addition, the concentration of 1000 μg/L induced hyperactivity (*p* < 0.01) during the third dark period (F_(4,135)_ = 3.5, *p* = 0.01). Taken together, there is evidence that TBH induces hyperactivity in zebrafish larvae, suggesting neurotoxicity or impaired motor movements.

### 3.3. TBH Reduced ROS Levels in Larval Zebrafish

**Figure 5:**
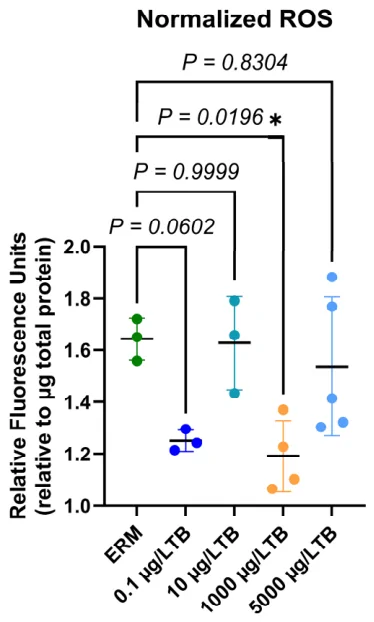
Whole-body reactive oxygen species (ROS) production in zebrafish larvae (5 dpf) following exposure to TBH. ROS levels are expressed as relative fluorescence units normalized to total protein content (µg protein/mL media). Data are presented as mean ± SD (*n* = 3–5 biological replicates of 10–15 larvae per treatment). Asterisks indicate a significant difference compared to the control group (*p* < 0.05), as determined by a One-Way ANOVA followed by Dunnett’s multiple comparisons test.

ROS levels were significantly reduced in larvae exposed to 1000 μg/L TBH compared to the ERM control (F_(4,13)_ = 4.82, *p* = 0.013). A similar response was observed at 0.1 μg/L TBH; however, this response did not reach statistical significance (*p* = 0.0602). No differences in ROS levels were detected in the remaining groups (Figure 5).

### 3.4. TBH Did Not Induce Apoptosis in Larval Fish Based on AO Staining

**Figure 6:**
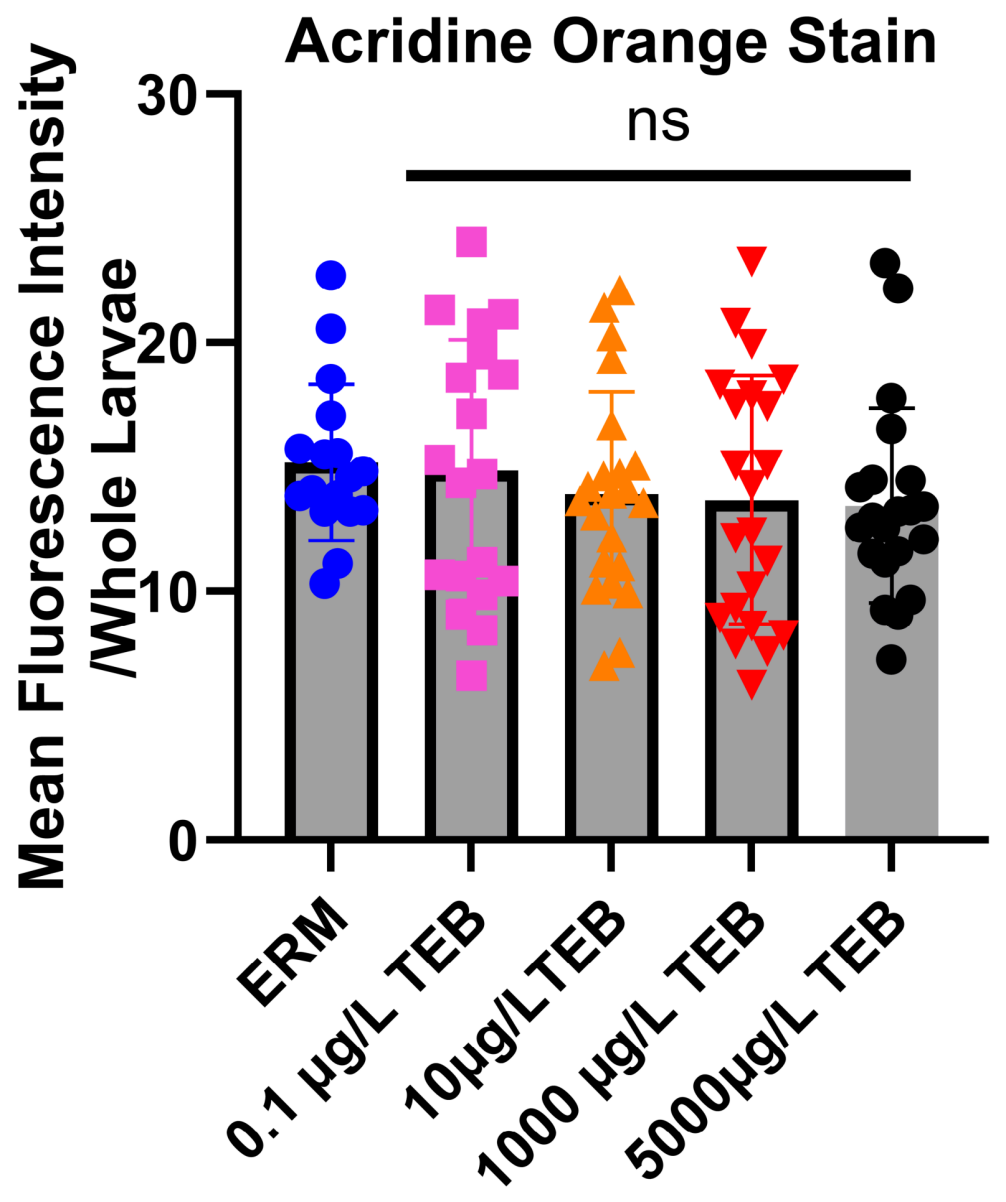
Effect of TBH on whole-body apoptosis in zebrafish larvae. Mean fluorescence intensity of Acridine Orange (AO) stain in 5 dpf larvae exposed to Embryo Rearing Media (ERM control) or varying concentrations of TBH (0.1 up to 5000 μg/L), (*n* = 17–22 per treatment). Data are presented as mean ± SD. “ns” indicates no statistical significance among treatment groups compared to the control (One-Way ANOVA followed by Dunnett’s multiple test relative to ERM).

Exposure to TBH did not induce significant alterations in whole-body apoptosis in 5 dpf zebrafish larvae. Quantification of acridine orange (AO) staining revealed no statistically significant differences in mean fluorescence intensity across any of the tested concentrations (0.1, 10, 1000, and 5000 μg/L), (F_(4,96)_ = 0.58, *p* = 0.68) relative to the ERM negative control (Figure 6).

### 3.5. TBH Altered the Expression of Select Genes Involved in Oxidative Stress and Neurotoxicity

Transcriptional analysis revealed alterations in the expression of genes associated with oxidative stress, apoptosis, and neurodevelopment. Rather than exhibiting a simple concentration-dependent linear response, the expression data demonstrated effects on transcripts at specific exposure thresholds.

**Figure 7:**
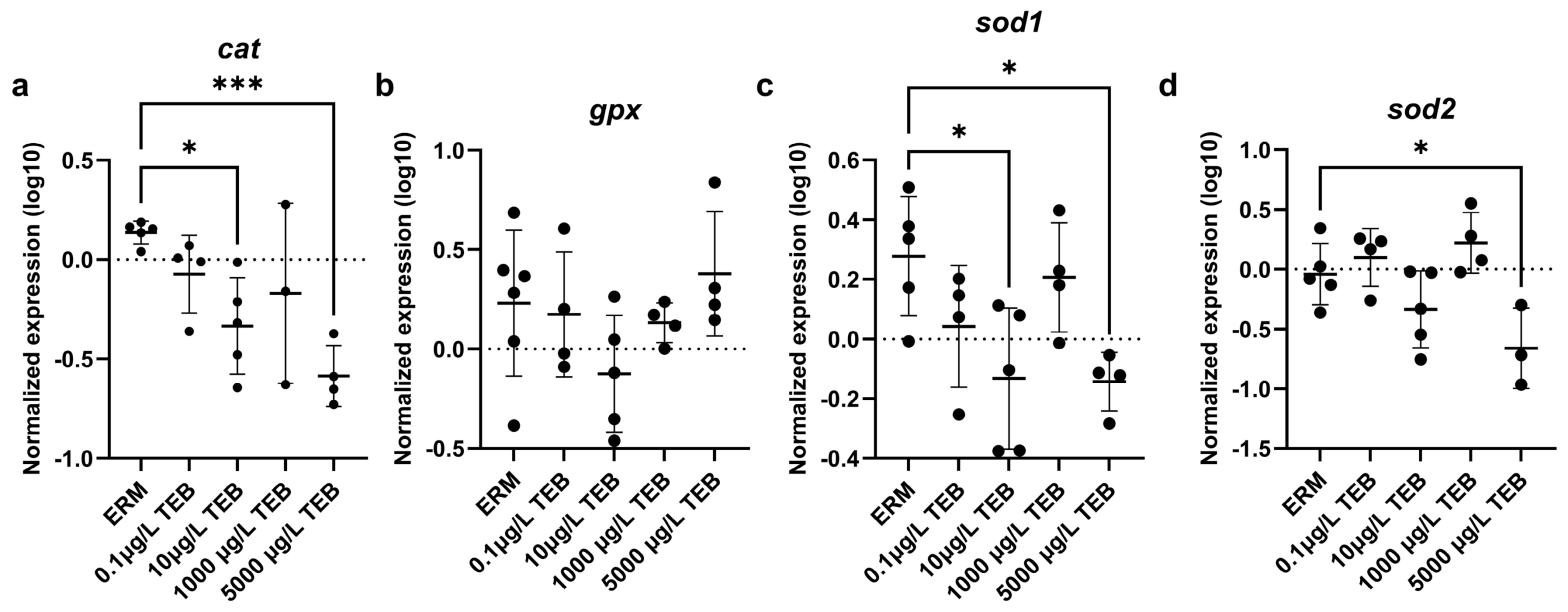
Transcriptional responses in 5 dpf zebrafish larvae following continuous TEB exposure. Normalized relative expression (log10) of targeted genes related to oxidative stress: (**a**) *cat*, (**b**) *gpx*, (**c**) *sod1*, and (**d**) *sod2*. The expression profiles highlight distinct statistical anomalies and non-linear concentration–response patterns. Data are expressed as mean ± SD. Each dot is a biological replicate. Asterisks denote statistical significance compared to the ERM control group (* *p* < 0.05, *** *p* < 0.001; One-Way ANOVA followed by Dunnett’s multiple comparisons test), *n* = (3–6).

**Figure 8:**
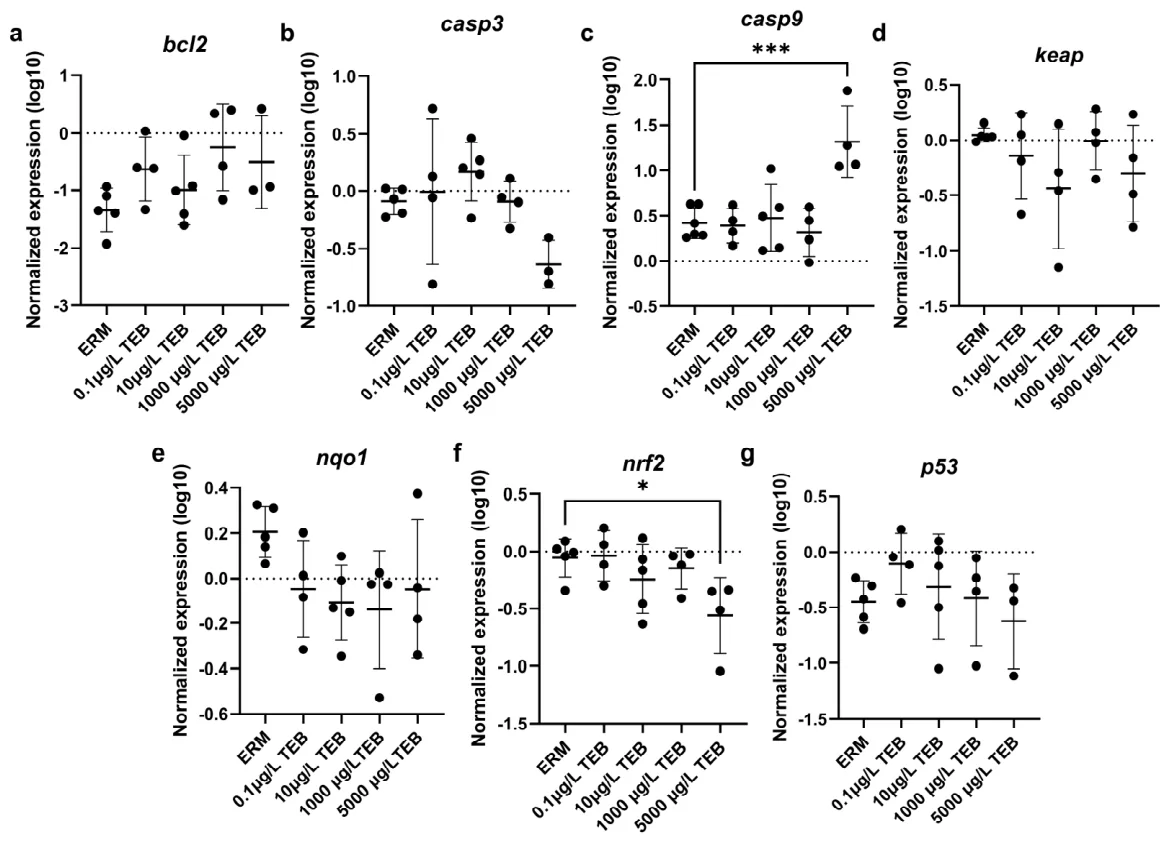
Transcriptional responses in 5 dpf zebrafish larvae following continuous TEB exposure. Normalized relative expression (log10) of targeted genes related to apoptosis: (**a**) *bcl2*, (**b**) *casp3*, (**c**) *casp9*, (**d**) *keap*, (**e**) *nqo1*, (**f**) *nrf2*, (**g**) *p53*. The expression profiles highlight distinct statistical anomalies and non-linear concentration–response patterns. Data are expressed as mean ± SD. Asterisks denote statistical significance compared to the ERM control group (* *p* < 0.05, *** *p* < 0.001; One-Way ANOVA followed by Dunnett’s multiple comparisons test), *n* = (3–6).

**Figure 9:**
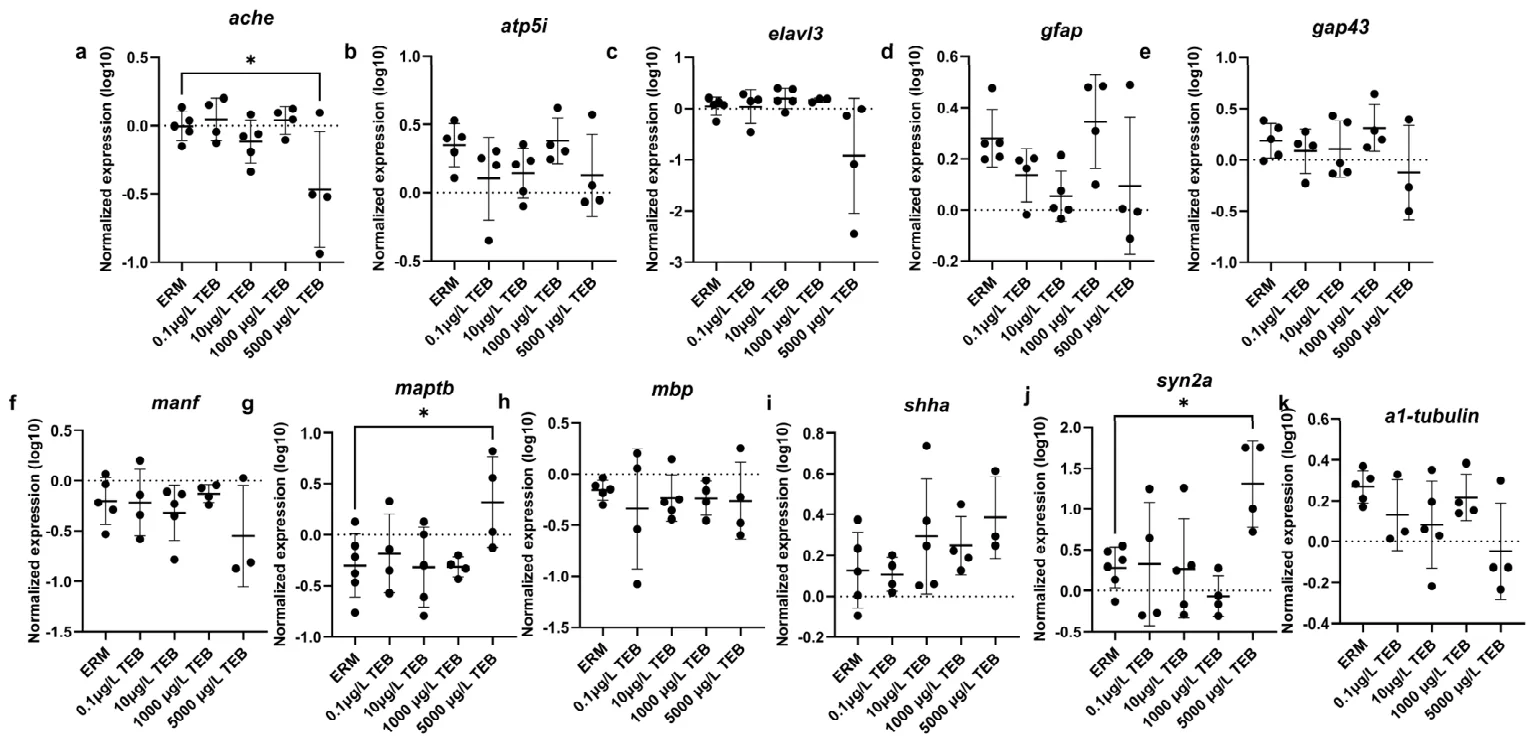
Transcriptional responses in 5 dpf zebrafish larvae following continuous TEB exposure. Normalized relative expression (log10) of targeted genes related to neurotoxicity: (**a**) *ache*, (**b**) *atp5i*, (**c**) *elavl3*, (**d**) *gfap*, (**e**) *gap43*, (**f**) *manf*, (**g**) *maptb*, (**h**) *mbp*, (**i**) *shha*, (**j**) *syn2a*, and (**k**) *alpha 1 tubulin*. The expression profiles highlight distinct statistical anomalies and non-linear concentration–response patterns. Data are expressed as mean ± SD. Asterisks denote statistical significance compared to the ERM control group (* *p* < 0.05; One-Way ANOVA followed by Dunnett’s multiple comparisons test), *n* = (3–6).

Regarding oxidative stress markers, *cat* expression was significantly downregulated (F_(4,16)_ = 6.24; *p* = 0.0032) at both 10 μg/L (*p* < 0.05) and 5000 μg/L (*p* < 0.001) (Figure 7a). Similarly, *sod1* exhibited significant downregulation (F_(4,17)_ = 4.492; *p* = 0.012) at 10 μg/L (post hoc *p* = 0.013) and 5000 μg/L (post hoc *p* = 0.017) (Figure 7c), whereas *sod2* showed a significant decrease (F_(4,16)_ = 5.64; *p* = 0.005) only at 5000 μg/L (post hoc *p* = 0.028) (Figure 7d). A corresponding change was observed in the intrinsic apoptotic pathway, with *casp9* demonstrating highly significant upregulation (F_(4,18)_ = 8.569; *p* = 0.0005) at 5000 μg/L (post hoc *p* = 0.001) (Figure 8c) and downregulation of *nrf2* (F_(4,17)_ = 3.04, *p* = 0.046) at 5000 μg/L (post hoc *p* = 0.025) (Figure 8f). Markers for neurodevelopment and synaptic function also displayed anomalous expression peaks; both *maptb* (F_(4,18)_ = 2.60; *p* = 0.071) at 5000 μg/L (post hoc *p* = 0.043) (Figure 9g) and *syn2a* (F_(4,18)_ = 4.32; *p*= 0.013) at 5000 μg/L (post hoc *p* = 0.019) (Figure 9j) were significantly upregulated. Conversely, cholinergic signaling was disrupted at a different threshold, with *ache* expression being significantly suppressed (F_(4,17)_ = 4.02; *p*= 0.018) at 5000 μg/L (*p* = 0.02) (Figure 9a). Other evaluated transcripts, *atp5i* (F_(4,17)_ = 1.50, *p* = 0.25), *bcl2* (F_(4,16)_ = 1.95, *p* = 0.15), *casp3* (F_(4,16)_ = 3, *p* = 0.051), *elavl3* (F_(4,16)_ = 3.20, *p* = 0.041), *gap43* (F_(4,16)_ = 1.23, *p* = 0.34), *gfap* (F_(4,17)_ = 2.68, *p* = 0.067), *gpx* (F_(4,18)_ = 1.73, *p* = 0.19), *keap* (F_(4,16)_ = 1.31, *p* = 0.31), *manf* (F_(4,16)_ = 1.06, *p* = 0.41), *mbp* (F_(4,17)_ = 0.18, *p* = 0.95), *nqo1* (F_(4,17)_ = 2.22, *p* = 0.11), *p53* (F_(4,17)_ = 0.98, *p* = 0.4), *shha* (F_(4,16)_ = 1.33, *p* = 0.31), and *a1 tubulin* (F_(4,16)_ = 1.64, *p* = 0.21), did not show significant differential expression across the evaluated concentrations.

## 4. Discussion

The toxicological profile established in this study indicates that the herbicide TBH acts primarily at physiological and biochemical levels, rather than as a structural teratogen. The lack of acute lethality, cardiotoxicity, and gross teratogenicity observed at concentrations up to 5000 μg/L strongly corroborates the baseline developmental observations previously reported for TBH in zebrafish embryos [15]. From a toxicological standpoint, these stable physiological milestones are highly informative; they suggest that subsequent behavioral and transcriptomic alterations are specific molecular responses to the herbicide, rather than secondary consequences of a systemic physiological collapse. Similar findings have been documented for another phenylurea herbicide, linuron, where early developmental stages and heart rates remained largely unaffected at concentrations up to 623 µg/L and 4982 µg/L, respectively, reinforcing the premise that phenylurea-induced toxicity predominantly targets molecular pathways over structural integrity [20].

Despite the absence of morphological defects, TBH exposure elicited significant, irregular behavioral alterations in 5 dpf larvae, characterized by an altered photomotor response. The Visual Motor Response (VMR) assay captures the integration of sensory processing and motor execution; because larval locomotion is highly dependent on oxidative respiration efficiency, xenobiotic-induced metabolic reductions can manifest hypoactivity and a failure to habituate to photic transitions [22]. While the initial sudden transition to darkness (Dark 1) did not yield significant deviations from the control, a paradoxical hyperactive response emerged during the subsequent photic phases. Sudden transitions to light naturally induce basal hypoactivity in healthy zebrafish; however, larvae exposed to 10 μg/L TBH exhibited pronounced hyperactivity during the Light 1 phase. This anomalous hyper-arousal persisted and expanded during the Light 2 phase, manifesting significantly in both the 10 and 1000 μg/L exposure groups. Furthermore, this failure to habituate eventually cascaded into the dark phases, with the 1000 μg/L concentration inducing significant hyperactivity during the final Dark 3 period. While de Oliveira et al. [15] previously documented persistent hyperactivity specifically during dark periods in older (6 dpf) larvae exposed to TBH, the current assessment uncovers a developmentally earlier and distinct photomotor shift, defined by light-induced hyperactivity that eventually disrupts dark-phase rest. This trajectory suggests that TBH actively dysregulates the larvae’s ability to acclimate to dynamic lighting conditions. Notably, this hyperactive manifestation contrasts with the behavioral phenotype reported for the structurally related herbicide linuron, which induced profound hypoactivity under similar VMR paradigms [20]. This underscores that while phenylureas can disrupt sensory–motor integration, their specific behavioral manifestations can be highly distinct.

We hypothesize that these early-stage behavioral shifts may be linked to underlying metabolic or oxidative stress. Typically, xenobiotic exposure triggers an ROS generation burst that overwhelms endogenous antioxidant defenses [37]. However, TBH exposure did not induce a traditional oxidative burst; instead, the reduction in ROS levels observed at 1000 μg/L, together with the comparable but non-significant response detected at 0.1 μg/L, may suggest a non-monotonic response pattern. However, additional studies are required to confirm this observation. The balance of ROS is dependent upon many factors, such as the activity of antioxidant enzyme expression and activity. A low dose may stimulate initial activity, which drives down ROS, while a high dose of TBH exposure leads to a ramping up of protective mechanisms for oxidative stress. The two intermediate concentrations may not have resulted in such a vigorous response, or the antioxidants were sufficient to keep ROS levels at basal levels at these exposure concentrations. Nevertheless, this intriguing non-monotonic response in ROS levels requires further investigation.

This depletion correlates with the pronounced hypoactivity observed in the exact same treatment group during the photic phase of the VMR assay. While this reduction could theoretically suggest a suppression in mitochondrial bioenergetics—which would directly reduce the natural byproduct of basal ROS—decreased H2DCFDA fluorescence might also stem from alternative changes in cellular metabolism or altered antioxidant scavenging activity. Recent toxicological assessments of other phenylurea herbicides, such as linuron and diuron, have demonstrated mitochondrial dysfunction and disrupted oxidative phosphorylation in early developmental stages [38,39,40]. If a similar mechanism occurs with TBH, such mitochondrial suppression may limit ATP availability, severely compromising the larvae’s capacity to maintain normal photomotor responses at 5 dpf. However, without complementary measurements of mitochondrial physiology, the precise impact of TBH on bioenergetic pathways remains speculative and warrants direct functional investigation. Interestingly, at the highest concentration (5000 μg/L), ROS levels were statistically indistinguishable from the control. This further illustrates the irregular concentration–response pattern, suggesting that at extreme toxicity thresholds, secondary stress pathways, such as endoplasmic reticulum (ER) stress or the onset of inflammatory responses, might generate compensatory ROS, masking the underlying depletion.

The transcriptional profiling of 5 dpf zebrafish larvae exposed to TBH revealed highly specific alterations that challenge the classical dose-dependent paradigm. Regarding oxidative stress, the significant downregulation of *cat* at 10 and 5000 μg/L, alongside the isolated suppression of *sod1* (10 and 5000 μg/L) and *sod2* (5000 μg/L), reveals an irregular concentration–response pattern. Rather than mounting a sustained, linear antioxidant defense, the downregulation of these critical enzymes at specific thresholds suggests a potential early exhaustion or impairment of the endogenous adaptive pathways at the transcript level. This hypothesis of systemic antioxidant collapse is strongly corroborated by the significant downregulation of *nrf2a* exclusively at the highest concentration (5000 μg/L). The *nrf2a* transcription factor serves as the master regulator of the cellular antioxidant response, responsible for driving the expression of downstream detoxifying enzymes like SOD and catalase [41]. Its transcriptional suppression at peak exposure levels suggests that TBH may impair the upstream regulatory machinery necessary to combat oxidative damage. Similar irregular transcriptional profiles and antioxidant fatigue have been documented in zebrafish exposed to other widespread contaminants, such as the PSII inhibitor atrazine [42,43] and dimethyl phthalate [44]. This lack of a compensatory transcriptional response aligns with the profound basal metabolic depression observed in the ROS and VMR assays, suggesting that TBH dysregulates energy allocation, which could theoretically precipitate the fatigue of the antioxidant defense system before a linear response can be fully mounted. However, as these observations are strictly based on mRNA abundance, complementary measurements of protein levels and antioxidant enzyme activities are required to validate functional exhaustion.

This state of physiological stress also appeared to trigger compensatory mechanisms within the developing nervous system. Consistent with de Oliveira et al. [15], who reported that TBH exposure did not significantly affect the mRNA levels of four key neurodevelopmental marker genes (*elavl3*, *gfap*, *gap43*, and *shha*), the basal neurodevelopmental transcripts evaluated in the present study similarly remained unaltered. However, the significant upregulation of *maptb* and *syn2a* exclusively at the highest concentration (5000 μg/L) suggests an acute hyper-compensatory response. The *maptb* gene encodes for tau proteins essential for axonal microtubule stabilization [45], while *syn2a* is crucial for anchoring presynaptic vesicles [46]. The sudden increase in the transcription of these specific structural markers may reflect an active attempt by the larval nervous system to preserve cellular connectivity and stabilize disrupted synaptic architectures in the face of impending xenobiotic neurotoxicity, a transcriptional defense mechanism similarly observed under developmental neurotoxicity induced by difenoconazole [29], trichlorfon [47], and flame retardants [48,49]. This localized transcriptional response may provide a molecular context for the atypical hyperactive motor responses observed during the light phases of the behavioral assay. Furthermore, this neurological dysregulation is corroborated by the significant transcriptional suppression of *ache* at 5000 μg/L. The downregulation of this critical cholinergic transcript at 5 dpf provides molecular support for the hypothesis that the late-stage AChE enzymatic inhibition observed by de Oliveira et al. [15] at concentrations above 100 μg/L may originate as a transcriptional failure during earlier developmental windows.

Perhaps the most counterintuitive finding of this assessment is the discrepancy between the intrinsic apoptotic pathway transcription and phenotypic cell death. Real-time PCR revealed a significant upregulation of *casp9* at 5000 μg/L; however, in vivo whole-body apoptosis assays using Acridine Orange (AO) staining showed no significant phenotypic evidence of cell death. There are several plausible explanations for this transcriptional–phenotypic decoupling. First, it may reflect a temporal latency; the sensitivity of qPCR captures early shifts in mRNA that precede the execution of apoptosis, which may not manifest phenotypically by 5 dpf. Alternatively, this could represent a transient transcriptional stress response that does not ultimately result in protein-level activation or widespread cell death. Beyond these simpler explanations, it is also biologically plausible, though entirely speculative at this stage, that this upregulation serves as a non-apoptotic function. Sub-lethal and compartmentalized *casp9* activity is a known physiological requirement for non-apoptotic neurodevelopmental processes, specifically axon pruning, synapse elimination, and glial differentiation [50,51]. The concurrent upregulation of synaptic (*syn2a*) and microtubule (*maptb*) transcripts could theoretically hint at a localized neuro-centric remodeling mechanism. However, without functional protein quantification or active caspase measurements, the precise nature of this apoptotic transcriptional spike remains a hypothesis requiring further experimental validation.

The integration of behavioral, biochemical, and molecular endpoints in this study provides a comprehensive look at TBH ecotoxicity that fundamentally challenges classical, linear dose–response paradigms. While traditional macro-structural assessments could erroneously classify this herbicide as low-risk due to the absence of gross teratogenicity, this multi-tiered approach uncovers a complex sequence of sublethal responses. The data points toward a cascade of potential metabolic and oxidative shifts that may affect larval locomotion, alongside transcriptional evidence of neurodevelopmental stress and early apoptotic signaling at higher thresholds. These irregular response patterns highlight that evaluating pesticide safety based solely on lethality or visible malformations severely underestimates the silent, systemic physiological disruptions that phenylurea herbicides pose to non-target aquatic vertebrates.

## Data Availability

The original contributions presented in this study are included in the article/Supplementary Materials. Further inquiries can be directed to the corresponding author.

## Supplementary Materials

The following supporting information can be downloaded at: https://www.mdpi.com/article/10.3390/jox16050166/s1. Table S1. Sequences of primers used in RT-qPCR.

## Abbreviations

The following abbreviations are used in this manuscript:

ERM	Embryo Rearing Media
GFP	Green Fluorescent Protein
PBS	Phosphate-Buffered Saline
PCR	Polymerase Chain Reaction
TBH	Tebuthiuron
NMDR	Non-Monotonic Dose–Response
VMR	Visual Motor Response

## References

[1] Franco-Bernardes M.F., Rocha O.P., Pereira L.C., Tasso M.J., Meireles G., de Oliveira D.P., Dorta D.J. (2017). The herbicides trifluralin and tebuthiuron have no genotoxic or mutagenic potential as evidenced by genetic tests. Environ. Sci. Pollut. Res..

[2] Agência Nacional de Vigilância Sanitária (2013). Resolução da Diretoria Colegiada—RDC nº 36, de 25/07/2013.

[3] Zhang W. (2018). Global pesticide use: Profile, trend, cost/benefit and more. Proc. Int. Acad. Ecol. Environ. Sci..

[4] Moreira B.R.d.A., Cruz V.H., Barbosa Júnior M.R., de Vasconcelos L.G., da Silva R.P., Lopes P.R.M. (2024). Adsorption of tebuthiuron on hydrochar: Structural, kinetic, isothermal, and mechanistic modeling, and ecotoxicological validation of remediative treatment of aqueous system. Biomass Convers. Biorefin..

[5] de Almeida M.D., Pereira T.S.B., Batlouni S.R., Boscolo C.N.P., de Almeida E.A. (2018). Estrogenic and anti-androgenic effects of the herbicide tebuthiuron in male Nile tilapia (*Oreochromis niloticus*). Aquat. Toxicol..

[6] Mostafa F.I., Helling C.S. (2003). Isolation and 16S DNA characterization of soil microorganisms from tropical soils capable of utilizing the herbicides hexazinone and tebuthiuron. J. Environ. Sci. Health Part B.

[7] Qian Y., Matsumoto H., Liu X., Li S., Liang X., Liu Y., Zhu G., Wang M. (2017). Dissipation, occurrence and risk assessment of a phenylurea herbicide tebuthiuron in sugarcane and aquatic ecosystems in South China. Environ. Pollut..

[8] de Lima E.W., Brunaldi B.P., Frias Y.A., de Almeida Moreira B.R., da Silva Alves L., Lopes P.R.M. (2022). A synergistic bacterial pool decomposes tebuthiuron in soil. Sci. Rep..

[9] Mendes K.F., Maset B.A., Mielke K.C., de Sousa R.N., Martins B.A.B., Tornisielo V.L. (2021). Phytoremediation of quinclorac and tebuthiuron-polluted soil by green manure plants. Int. J. Phytoremediation.

[10] Pires F.R., Souza C.M., Silva A.A., Queiroz M.E.L.R., Procópio S.O., Santos J.B., Santos E.A., Cecon P.R. (2003). Seleção de plantas com potencial para fitorremediação de tebuthiuron. Planta Daninha.

[11] Grott S.C., Israel N., Lima D., Bitschinski D., Abel G., Alves T.C., da Silva E.B., de Albuquerque C.A., Mattos J.J., Bainy A.C. (2022). Influence of temperature on growth, development and thyroid metabolism of American bullfrog tadpoles (*Lithobates catesbeianus*) exposed to the herbicide tebuthiuron. Environ. Toxicol. Pharmacol..

[12] Machado C.S., Alves R.I., Fregonesi B.M., Tonani K.A., Martinis B.S., Sierra J., Nadal M., Domingo J.L., Segura-Muñoz S. (2016). Chemical contamination of water and sediments in the Pardo River, São Paulo, Brazil. Procedia Eng..

[13] Barizon R.R.M., Kummrow F., de Albuquerque A.F., Assalin M.R., Rosa M.A., Dutra D.R.C.d.S., Pazianotto R.A.A. (2022). Surface water contamination from pesticide mixtures and risks to aquatic life in a high-input agricultural region of Brazil. Chemosphere.

[14] Scott G.R., Sloman K.A. (2004). The effects of environmental pollutants on complex fish behaviour: Integrating behavioural and physiological indicators of toxicity. Aquat. Toxicol..

[15] de Oliveira A.Á.S., Vieira L.C., Dreossi S.C., Dorta D.J., Gravato C., da Silva Ferreira M.E., de Oliveira D.P. (2023). Integrating morphological, biochemical, behavioural, and molecular approaches to investigate developmental toxicity triggered by tebuthiuron in zebrafish (*Danio rerio*). Chemosphere.

[16] Souza E.L.C., Foloni L.L., Mantovani E.C., Teixeira Filho J. (2008). Comportamento do tebuthiuron em solo de cultivo de cana-de-açúcar utilizando lisímetro de drenagem modificado. Planta Daninha.

[17] Temple A.J. (1985). The Effects of Tebuthiuron on Aquatic Productivity. Master’s Thesis.

[18] Elizalde-Velázquez G.A., Herrera-Vázquez S.E. (2023). Zebrafish as model organism in aquatic ecotoxicology: Current trends and future perspectives. Zebrafish Research-An Ever-Expanding Experimental Model.

[19] Marlatt V.L., Martyniuk C.J. (2017). Biological responses to phenylurea herbicides in fish and amphibians: New directions for characterizing mechanisms of toxicity. Comp. Biochem. Physiol. Part C Toxicol. Pharmacol..

[20] Maharaj S., El Ahmadie N., Rheingold S., El Chehouri J., Yang L., Souders C.L., Martyniuk C.J. (2020). Sub-lethal toxicity assessment of the phenylurea herbicide linuron in developing zebrafish (*Danio rerio*) embryo/larvae. Neurotoxicology Teratol..

[21] Huang T., Souders C.L., Wang S., Ganter J., He J., Zhao Y.H., Cheng H., Martyniuk C.J. (2021). Behavioral and developmental toxicity assessment of the strobilurin fungicide fenamidone in zebrafish embryos/larvae (*Danio rerio*). Ecotoxicol. Environ. Saf..

[22] Perez-Rodriguez V., Souders C.L., Tischuk C., Martyniuk C.J. (2019). Tebuconazole reduces basal oxidative respiration and promotes anxiolytic responses and hypoactivity in early-staged zebrafish (*Danio rerio*). Comp. Biochem. Physiol. Part C Toxicol. Pharmacol..

[23] Ivantsova E., Konig I., Lopez-Scarim V., English C., Charnas S.R., Souders C.L., Martyniuk C.J. (2023). Molecular and behavioral toxicity assessment of tiafenacil, a novel PPO-inhibiting herbicide, in zebrafish embryos/larvae. Environ. Toxicol. Pharmacol..

[24] An G., Hong T., Park H., Lim W., Song G. (2023). Oxamyl exerts developmental toxic effects in zebrafish by disrupting the mitochondrial electron transport chain and modulating PI3K/Akt and p38 Mapk signaling. Sci. Total Environ..

[25] Lu X., Liu Y., Zhang D., Liu K., Wang Q., Wang H. (2021). Determination of the panel of reference genes for quantitative real-time PCR in fetal and adult rat intestines. Reprod. Toxicol..

[26] Deng J., Yu L., Liu C., Yu K., Shi X., Yeung L.W., Lam P.K., Wu R.S., Zhou B. (2009). Hexabromocyclododecane-induced developmental toxicity and apoptosis in zebrafish embryos. Aquat. Toxicol..

[27] Sarkar S., Mukherjee S., Chattopadhyay A., Bhattacharya S. (2014). Low dose of arsenic trioxide triggers oxidative stress in zebrafish brain: Expression of antioxidant genes. Ecotoxicol. Environ. Saf..

[28] Luzio A., Monteiro S.M., Fontaínhas-Fernandes A.A., Pinto-Carnide O., Matos M., Coimbra A.M. (2013). Copper induced upregulation of apoptosis related genes in zebrafish (*Danio rerio*) gill. Aquat. Toxicol..

[29] Yang Q., Deng P., Xing D., Liu H., Shi F., Hu L., Zou X., Nie H., Zuo J., Zhuang Z. (2023). Developmental neurotoxicity of difenoconazole in zebrafish embryos. Toxics.

[30] Dong M., Wang J., Liu Y., He Q., Sun H., Xu Z., Hong H., Lin H., Gao P. (2023). 3-bromocarbazole-induced developmental neurotoxicity and effect mechanisms in zebrafish. ACS EST Water.

[31] Zhao X., Gong L., Wang C., Liu M., Hu N., Dai X., Peng C., Li Y. (2021). Quercetin mitigates ethanol-induced hepatic steatosis in zebrafish via P2X7R-mediated PI3K/Keap1/Nrf2 signaling pathway. J. Ethnopharmacol..

[32] Wang J., Mo C., Tu P., Ning N., Liu X., Lin S., Muthulakshmi S., He Z., Zhang Y., Liu K. (2021). Developmental toxicity of Zishen Guchong Pill on the early life stages of Zebrafish. Phytomed. Plus.

[33] Gao J., Liu X., Wang B., Huang Y., Chen R., Yuan W., Luo Q., Lu H., Tian G. (2025). Acute toxicity effects of rice paddy bactericide bismerthiazol on zebrafish (*Danio rerio*) embryos. Comp. Biochem. Physiol. Part C Toxicol. Pharmacol..

[34] Wu Q., Yan W., Liu C., Li L., Yu L., Zhao S., Li G. (2016). Microcystin-LR exposure induces developmental neurotoxicity in zebrafish embryo. Environ. Pollut..

[35] Baronio D., Chen Y., Decker A.R., Enckell L., Fernández-López B., Semenova S., Puttonen H.A.J., Cornell R.A., Panula P. (2022). Vesicular monoamine transporter 2 (SLC18A2) regulates monoamine turnover and brain development in zebrafish. Acta Physiol..

[36] Chen T., Zhou L., Yuan Y., Fang Y., Guo Y., Huang H., Zhou Q., Lv X. (2014). Characterization of *Bbx*, a member of a novel subfamily of the HMG-box superfamily together with *Cic*. Dev. Genes Evol..

[37] Lushchak V.I. (2016). Contaminant-induced oxidative stress in fish: A mechanistic approach. Fish Physiol. Biochem..

[38] Jakaria M., Cannon J.R. (2025). Insights into cytotoxicity and redox modulation by the herbicide linuron and its metabolite, 3, 4-dichloroaniline. Neurotoxicology.

[39] Lima T.R.R., Kohori N.A., de Camargo J.L.V., da Silva C.A., Pereira L.C. (2024). Diuron and its metabolites induce mitochondrial dysfunction-mediated cytotoxicity in urothelial cells. Toxicol. Mech. Methods.

[40] Shi J., Luo T., Wang Z., Ren X., Ran Y., Peng Y., Liu G., Peijnenburg W. (2026). A metabolomics study unravels the hepatotoxic mechanism of diuron in zebrafish: Disruption of glutathione synthesis and mitochondrial energy metabolism. Aquat. Toxicol..

[41] Li H., Zhang Q., Li W., Li H., Bao J., Yang C., Wang A., Wei J., Chen S., Jin H. (2019). Role of Nrf2 in the antioxidation and oxidative stress induced developmental toxicity of honokiol in zebrafish. Toxicol. Appl. Pharmacol..

[42] Khoshnood Z. (2024). A review on toxic effects of pesticides in zebrafish, *Danio rerio* and common carp, *Cyprinus carpio*, emphasising atrazine herbicide. Toxicol. Rep..

[43] Jin Y., Zhang X., Shu L., Chen L., Sun L., Qian H., Liu W., Fu Z. (2010). Oxidative stress response and gene expression with atrazine exposure in adult female zebrafish (*Danio rerio*). Chemosphere.

[44] Cong B., Liu C., Wang L., Chai Y. (2020). The impact on antioxidant enzyme activity and related gene expression following adult zebrafish (*Danio rerio*) exposure to dimethyl phthalate. Animals.

[45] Nik S.H.M., Newman M., Ganesan S., Chen M., Martins R., Verdile G., Lardelli M. (2014). Hypoxia alters expression of Zebrafish Microtubule-associated protein Tau (*mapta*, *maptb*) gene transcripts. BMC Res. Notes.

[46] Pullaguri N., Grover P., Abhishek S., Rajakumara E., Bhargava Y., Bhargava A. (2021). Triclosan affects motor function in zebrafish larva by inhibiting ache and syn2a genes. Chemosphere.

[47] Shi Q., Yang H., Chen Y., Zheng N., Li X., Wang X., Ding W., Zhang B. (2023). Developmental neurotoxicity of trichlorfon in zebrafish larvae. Int. J. Mol. Sci..

[48] Sun Y., Wang X., Zhou S., Zhou Y., Hua J., Guo Y., Wang Y., Zhang W., Yang L., Zhou B. (2023). Evaluation and mechanistic study of transgenerational neurotoxicity in zebrafish upon life cycle exposure to decabromodiphenyl ethane (DBDPE). Environ. Sci. Technol..

[49] Zhang X., Guo L., Luo Y., Xu X., Han Y., Chen H., Sun H., Xue Y., Ji G. (2025). Neurotoxicity and Mechanism in Zebrafish Embryo Induced by Tetrabromobisphenol A bis (2-Hydroxyethyl) Ether (TBBPA-DHEE) Exposure. Toxics.

[50] Mukherjee A., Williams D.W. (2017). More alive than dead: Non-apoptotic roles for caspases in neuronal development, plasticity and disease. Cell Death Differ..

[51] Hollville E., Deshmukh M. (2018). Physiological functions of non-apoptotic caspase activity in the nervous system. Semin. Cell Dev. Biol..

