# Supplementary material for: An Integrative Toxicological Assessment of the Herbicide Tebuthiuron: Elucidating Biochemical and Behavioral Responses in Developing Zebrafish
Source: J Xenobiot. 2026 Sep 3;16(5):166. doi: 10.3390/jox16050166 (PMC13608060; doi:10.3390/jox16050166)
Supplement: Supplementary file 1 [file jox-16-00166-s001.zip › jox-4464363-supplementary.pdf]

Supplementary Materials: An Integrative Toxicological Assessment of the Herbicide Tebuthiuron: Elucidating Biochemical and Behavioral Responses in Developing Zebrafish

Giovane Ferreira, Amany Sultan, Fatma Ceren Kirgiz, Jacqueline Gutierrez, Evelyn C. López González, Christopher J. Martyniuk

Table S1. Sequences of primers used in RT-qPCR.

Gene name	Gene Symbol	Forward (5' to 3')	Reverse (5' to 3')	Reference
Acetylcholin-esterase	*ache*	GCTAATGAGCAAAAGCATGTGGGC	TATCTGTGATGTTAAGCAGACGAGGCA	NM_131846.2 [23] Ivantsova et al., 2023
ATP synthase F1 subunit	*atp5i*	TCGGTCAGACAACAGTCAGG	CCTCTCTTCCCAATGATCTCC	NM_199757.1 [24] An et al., 2023
Beta-actin	*bactin*	GGACCTGACAGACTACCTCA	GTTGCCAATAGTGATGACCT	NM_131031.2 [25] Lu et al., 2021
BCL2 Apoptosis Regulator	*bcl2*	TCACTCGTTCAGACCCTCAT	ACGCTTTCCACGCACAT	[26] Deng et al., 2009
Catalase	*cat*	CTCCTGATGTGGCCCGATAC	TCAGATGCCCGGCCATATTC	[27] Sarkar et al., 2014
Caspase 3	*casp3*	CCGCTGCCCATCACTA	ATCCTTTCACGACCATCT	[26] Deng et al., 2009
Caspase 9	*cas9*	CTGAGGCAAGCCATAATCG	AGAGGACATGGGAATAGCGT	NM_001007404.2 [28] Luzio et.al., 2013
ELAV Like RNA Binding Protein 3	*elavl3*	AGACAAGATCACAGGCCAGAGCTT	TGGTCTGCAGTTTGAGACCGTTGA	NM_131449.1 [29] Yang et al., 2023
Growth Associated Protein 43	*gap43*	TTAACGGAGGACCAGTGCAA	GTCCTGATCTCCAGCACACG	NM_131341.1 [29] Dong et al., 2023
Glial Fibrillary Acidic Protein	*gfap*	GGATGCAGCCAATCGTAAT	TTCCAGGTCACAGGTCAG	NM_131373.2 [30] Dong et al., 2023
Glutathione peroxidase	*gpx*	CCCTCTGTTTGCGTTCCTGA	TCTTGAATGGTTCCCCGTCC	BC164790.1 [27] Sarkar et al., 2014
Kelch-like ECH-associated protein	*keap*	CCAACGGCATAGAGGTAGTTAT	CCTGTATGTGGTAGGAGGGTT	[31] Zhao et al., 2021
Mesencephalic Astrocyte Derived Neurotrophic Factor	*manf*	AGATGGAGAGTGTGAAGTCTGTGTG	CAATTGAGTCGCTGTCAAAACTTG	NM_001076629 [29] Yang et al., 2023
Myelin Basic Protein	*mbp*	AATCAGCAGGTTCTTCGGAGGAGA	AAGAAATGCACGACAGGGTTGACG	BC115202.1 [29] Yang et al., 2023
Microtubule-associated protein tau b	*maptb*	AAGATCGGCTCCACTGAGAACC	GATCCAACCTTTGACTGGGCTT	XM_073944310.1
(NAD(P)H deshidrogenasa quinona 1	*nqo*	CGCGAGATGTTGCAGTTCAG	AAGTGCTCGGGATTCTGCAA	[31] Zhao et al., 2021

Nuclear Factor Erythroid 2-Like 2	*nrf2*	AAGCAGACGGAGGAGGAG	GGAGGTGTTCAGGCAAGG	[32] Wang et al. 2021
Tumor Protein P53	*p53*	GGGCAATCAGCGAGCAAA	ACTGACCTTCCTGAGTCTCCA	NM_131327.2 [26] Deng et al., 2009
Sonic Hedgehog Signaling Molecule	*shha*	TGTCCTCGACAACTCAACGG	TCCGTGTATATCCGCTGCAC	NM_131063.3 [33] Gao et al., 2025
Superoxide dismutase 1	*sod1 (Cu/Zn SOD)*	CAACACAAACGGCTGCATCA	TTTGCAACACCACTGGCATC	[27] Sarkar et al., 2014
Superoxide dismutase 2	*Sod2 (Mn - SOD)*	AGCGTGACTTTGGCTCATTT	ATGAGACCTGTGGTCCCTTG	[27] Sarkar et al., 2014
Synapsin IIa	*syn2a*	TGTAACGGAGTTGCAGTGGG	CAACCCTGAAGAGACGACCG	NM_001002597.2 [30] Dong et al., 2023
Alpha-1-tubulin	*tubulin*	AATCACCAATGCTTGCTTCGAGCC	TTCACGTCTTTGGGTACCACGTCA	NM_194388 [34] Wu et al., 2016
Ribosomal protein L13A	*rpl13a*	AGAGAAAGCGCATGGTTGTCC	GCCTGGTACTTCCAGCCAACTT	[35] Baronio et al., 2022
Ribosomal protein S18 (rps18)	*rps18*	GTAAACCCGTTGAACCCCATT	CCATCCAATCGGTAGTAGCG	XR_012396427.1 [36]
